# Utility of the BioFire^®^ FilmArray^®^ Pneumonia Panel Using Bronchial Washing Specimens: A Comparative Study with Conventional Culture

**DOI:** 10.3390/diagnostics16010091

**Published:** 2025-12-26

**Authors:** Sungjin Jo, Sei Won Kim, Jung Ok Kim, Sang-hyun Shin, Sehee Kim, Heayon Lee, Chang Dong Yeo, Sang Haak Lee, In Young Yoo, Yeon-Joon Park

**Affiliations:** 1 Department of Laboratory Medicine, Eunpyeong St. Mary’s Hospital, College of Medicine, The Catholic University of Korea, Seoul 03312, Republic of Korea; 21100379@cmcnu.or.kr (S.J.); wjddhr911@naver.com (J.O.K.);; 2 Division of Pulmonary, Department of Internal Medicine, Eunpyeong St. Mary’s Hospital, College of Medicine, The Catholic University of Korea, Seoul 03312, Republic of Korea; 3 Department of Laboratory Medicine, Seoul St. Mary’s Hospital, College of Medicine, The Catholic University of Korea, Seoul 06591, Republic of Korea

**Keywords:** pneumonia, FilmArray Pneumonia Panel, bronchial washing, multiplex PCR

## Abstract

**Background:** Accurate identification of pneumonia pathogens is critical for guiding appropriate antibiotic therapy and minimizing unnecessary antimicrobial exposure. Bronchoalveolar lavage (BAL) is widely used for pathogen detection but introduces procedural risks. Bronchial washing (BW) is simpler and less invasive, yet evidence for its utility in multiplex PCR diagnostics is limited. **Methods:** This study includes an evaluation of the clinical utility of the BioFire^®^ FilmArray^®^ Pneumonia Panel (FA) using BW specimens via comparison with conventional culture. Between 2022 and 2024, 190 BW specimens were collected from 182 adult patients with suspected pneumonia at Eunpyeong St. Mary’s Hospital, Seoul, Korea. Each specimen was tested simultaneously using FA and conventional culture. **Results:** The culture positivity rate was 41.6%, whereas FA showed a higher positivity rate of 51.1%. Of all specimens, 52.6% (100/190) were positive in at least one of two methods, and 11.0% (21/190) were FA-positive only. FA detected 72 additional bacterial targets, most commonly *H. influenzae*, *K. pneumoniae*, *S. aureus*, *S. agalactiae*, and *S. pneumoniae*. Semi-quantitative results demonstrated a statistically significant moderate correlation with culture (ρ = 0.48, *p* < 0.001). Eight bacterial targets achieved 100% PPA, and resistance genes were rapidly detected, although some discrepancies with phenotypic antimicrobial susceptibility testing were observed. Several FA-only detections may reflect oropharyngeal colonization rather than true infection. **Conclusions:** FA testing of BW specimens demonstrated high concordance with culture and provided rapid pathogen and resistance gene detection. BW-based FA testing may serve as a useful diagnostic alternative when BAL is not feasible, although careful interpretation is required to account for potential contamination.

## 1. Introduction

Identification of the causative pathogen is critical for effective treatment of pneumonia, as based on the laboratory findings, it guides appropriate antibiotic therapy and minimizes unnecessary antimicrobial exposure [1]. Accurate identification of pathogens in pneumonia is associated with reduced mortality, largely due to the implementation of targeted therapy [2,3].

Conventional culture remains essential for establishing definitive antimicrobial treatment. However, some pathogens are difficult to cultivate, and factors such as microbial growth characteristics of commensal flora overgrowth may hinder consistent pathogen identification [4]. In addition, atypical bacteria and viruses require supplementary diagnostic methods beyond culture, which clinicians may not routinely order, thereby limiting the efficacy of targeted therapy [5].

Bronchoalveolar lavage (BAL) is used in cases of unresolved pneumonia, but the procedure risks reducing oxygen saturation in patients [6,7]. In contrast, bronchial washing (BW) uses a smaller volume of lavage fluid, resulting in a shorter procedure time and lower risk of oxygen desaturation. Culture-based identification of bacterial pathogens in pneumonia has shown substantial concordance between BAL and BW specimens [8]. Although studies on the diagnostic performance of multiplex PCR using BW specimens remain limited, viral detection using PCR has demonstrated high concordance between BAL and BW samples [9,10].

The BioFire^®^ FilmArray^®^ Pneumonia Panel (FA; BioFire Diagnostics LLC., Salt Lake City, UT, USA) is a sample-to-result PCR system that semi-quantitatively reports 15 bacterial targets and qualitatively detects viruses, atypical pneumonia bacteria, and antibiotic resistance genes, and has been validated for use with specimens such as sputum, BAL, and mini-BAL. The semi-quantitative output of FA aligns with culture-based reporting protocols, aiding in the distinction between true pathogens and colonizers [5,11]. Clinical guidelines define significant infection thresholds as ≥10^4^ CFU/mL for BAL and ≥10^5^–10^6^ CFU/mL for endotracheal aspirates, helping reduce unnecessary antibiotic use for low-level colonizers. Similarly, FA employs detection thresholds to limit reporting of low-abundance bacteria [5].

However, comparative studies evaluating culture and multiplex PCR for pathogen identification using BW specimens are scarce. The aim of this study is to assess whether BW specimens are suitable for FA testing by comparing FA results with conventional culture findings, thereby evaluating the clinical utility of BW as a valid specimen type for molecular pneumonia diagnostics.

## 2. Materials and Methods

### 2.1. Study Population

This study included 182 adult patients who underwent bronchoscopy with BW at Eunpyeong St. Mary’s Hospital (Seoul, Republic of Korea) between 2022 and 2024 due to suspected pneumonia. Both conventional bacterial culture and the FA were simultaneously performed on BW specimens collected during bronchoscopy. All patients had strong clinical suspicion of pneumonia, and bronchoscopy was performed within 2 to 3 days after new radiologic findings, such as focal consolidation, ground-glass opacity, nodular or tree-in-bud opacities, or segmental infiltrates, were identified. Bronchoscopy was particularly considered when sputum studies failed to reveal a pathogen or when BAL was deemed high-risk, such as in mechanically ventilated patients. The procedure was conducted by experienced pulmonologists who targeted radiologically suspicious sites. Normal saline was instilled until at least 10 mL of aspirate was collected in a sterile trap bottle, with the total instilled volume not exceeding 50 mL. The initial 10 mL of aspirated fluid following saline instillation was defined as the BW sample to represent bronchial rather than alveolar sampling. All BW specimens were transported to the laboratory within 2 h after collection, and both FA and culture processing were initiated promptly without delay. Patients were excluded if the BW volume was insufficient for both FA and culture testing due to technical issues during bronchoscopy, such as poor suction recovery or early termination of the procedure. No additional exclusion criteria were applied.

This study was approved by the Institutional Review Board of Eunpyeong St. Mary’s Hospital (IRB No. XC21DIDI0136), with a waiver of informed consent for use of residual specimens and de-identified medical records. Patient demographic data, radiological findings, and hospitalization status were extracted from the electronic medical records (EMR) by a physician blinded to laboratory results.

### 2.2. BioFire FilmArray Pneumonia Panel (FA)

The FA test was performed according to the manufacturer’s instructions. BW specimens were processed in a biosafety cabinet. After vortexing, 100 μL of the specimen was mixed with the provided sample buffer (Catalog No. FAIV-GEN-0016) and loaded into the FA pouch, which was then inserted into the instrument. The FA panel semi-quantitatively reports bacterial targets with bin values corresponding to 10^4^, 10^5^, 10^6^, or ≥10^7^ copies/mL. Resistance genes are reported qualitatively only when their corresponding bacterial species are detected. Viruses and atypical bacteria are reported qualitatively as “detected” or “not detected.”

### 2.3. Conventional Culture

Following the removal of 100 μL for FA testing, the remaining specimen was processed for standard culture. After vortexing, 250 μL was centrifuged at 1,500 rpm for 5 min. The resulting pellet was used for Gram staining and inoculation. Cultures were plated on 5% sheep blood agar, CHOC-VBC agar, and MacConkey agar, and incubated at 35 °C with 5% CO_2_ for 48 h. Bacterial isolates were identified using matrix-assisted laser desorption ionization time-of-flight mass spectrometry (MALDI-TOF MS; Bruker Biotyper, Bruker Daltonics, Bremen, Germany). The quantity of growth was semi-quantitatively reported as “heavy,” “moderate,” or “slight” based on routine quadrant-streak evaluation, following standard clinical microbiology practice as described in *the Clinical Microbiology Procedures Handbook* [12]. Bronchial washing specimens do not have established CFU-based grading criteria; therefore, semi-quantitative streak-based interpretation was used. Normal oral flora in BW specimens was reported as “No pathogen”. Because BW was collected under bronchoscopic guidance, sputum-type rejection criteria and contamination scoring were not applied. Antimicrobial susceptibility testing (AST) was performed using either the MicroScan WalkAway system (Beckman Coulter, Brea, CA, USA) or Vitek 2 (bioMérieux, Marcy-l’Étoile, France). For isolates that exhibited resistance to carbapenems in AST, additional testing for carbapenemase genes was performed using either the Xpert Carba-R assay (Cepheid, Sunnyvale, CA, USA) or the BD MAX^TM^ Check-Points CPO assay (Checkpoints, Wageningen, The Netherlands).

### 2.4. Statistical Analysis

The concordance between FA and conventional culture was evaluated using positive percent agreement (PPA), negative percent agreement (NPA), and overall percent agreement (OPA), each with 95% confidence intervals (95% CI). To compare the semi-quantitative bacterial load results between FA and culture, Spearman’s rank correlation coefficient (ρ) was calculated. The culture categories (“slight”, “moderate”, “heavy”) were converted into ordinal ranks, which were compared with FA semi-quantitative bins. The strength of correlation was interpreted as follows: |ρ| ≥ 0.7: strong; 0.4 ≤ |ρ| < 0.7: moderate; 0.1 ≤ |ρ| < 0.4: weak; |ρ| < 0.1: none. A post hoc sample size calculation was conducted to justify the precision of the agreement estimates. Assuming an expected PPA and NPA of 90%, a 95% confidence level, and a margin of error of 0.10, the minimum number of specimens required for estimating each measure was calculated as 35 using the formula n = Z^2^ × P × (1 − P)/d^2^.

Statistical calculations, including PPA/NPA/OPA and confidence intervals, were performed using Microsoft Excel (Microsoft Corp., Redmond, WA, USA).

## 3. Results

### 3.1. Demographics

A total of 182 patients contributed 190 BW specimens to this study. Among them, 8 patients provided two specimens each, while the remaining 174 patients submitted one specimen each. Eight patients contributed two BW specimens due to repeat bronchoscopy performed on additional lobes or persistent clinical suspicion. Analyses were conducted at the specimen level. The median age of the patients was 74.0 years (±9.4, interquartile range: 63.3–82.0), and 132 (72.5%) were male. Most procedures were performed in the intensive care unit or bronchoscopy suite. Radiologic evidence of pulmonary infiltrates was present in 153 patients (84.1%), while 9 patients had chronic obstructive pulmonary disease (COPD), and 8 had lung cancer. In patients without definite infiltrates, bronchoscopy was performed based on clinical deterioration and nondiagnostic sputum studies. At the time of bronchoscopy, 180 of 182 patients (98.9%) were hospitalized, 65.9% (120/182) were admitted to the ICU, and 37.9% (69/182) required mechanical ventilation.

### 3.2. Pathogen Detection via FA vs. Conventional Culture

The number of bacterial pathogens detected per specimen using conventional culture and the FA test is summarized in Table 1. The culture positivity rate was 41.6% (79/190), with a total of 98 bacteria identified, corresponding to a mean of 0.52 isolates per specimen. In contrast, the FA yielded a higher positivity rate of 51.1% (97/190), detecting 164 bacterial targets overall, with a mean of 0.86 targets per specimen. Among the 190 BW specimens, 100 (52.6%) were positive for at least one bacterial pathogen by either FA or culture. An additional 21 specimens (11.0%) were positive only by FA, while 3 specimens (1.6%) were culture-positive for FA panel targets but negative by FA. Among specimens that were positive only by FA, the individual pathogen targets generally showed low FA bin value, with 14 targets at 10^4^ copies/mL, 4 at 10^5^ copies/mL, 4 at 10^6^ copies/mL, and only 1 target with a load ≥10^7^ copies/mL.

In total, 92 bacterial isolates were concurrently identified using both methods, whereas 72 additional bacterial targets were detected exclusively using FA. The most common bacterial pathogens detected only by FA were *H. influenzae* (*n* = 10), *K. pneumoniae* (*n* = 9), *S. aureus* (*n* = 15), *S. agalactiae* (*n* = 9), and *S. pneumoniae* (*n* = 8). These FA-only detections were predominantly associated with low-to-moderate semi-quantitative bin value (10^4^–10^6^ copies/mL), although a few reached ≥10^7^ copies/mL (Table 2). Conversely, six bacterial isolates were detected only by culture, despite being included in the FA panel targets; all were reported at low abundance. Specifically, one *K. aerogenes* isolate was reported as “moderate”, while two *K. pneumoniae*, two *P. aeruginosa*, and one *E. hormaechei* isolates were reported as “slight”.

The PPA, NPA, and OPA between FA and culture for each bacterial target are summarized in Table 3. Eight of the fifteen bacterial targets demonstrated 100% PPA with culture. *E. cloacae* complex and *K. aerogenes* showed comparatively lower PPA. Most targets demonstrated NPA values exceeding 97%. The OPA was greater than 90% for all targets.

### 3.3. Semi-Quantitative Comparison of FA and Conventional Culture

A comparison of the semi-quantitative bacterial load reported by FA and culture is presented in Table 2. A statistically significant moderate positive correlation was observed between the two methods (Spearman’s ρ = 0.48, *p* < 0.001). Among specimens with negative culture results, FA most frequently reported bacterial load at the 10^4^ copies/mL level (*n* = 36). Additionally, 14 isolates were reported at 10^5^ copies/mL and 13 at 10^6^ copies/mL. Notably, in 9 isolates, the bacterial load reported when using FA reached ≥10^7^ copies/mL.

### 3.4. Antimicrobial Resistance Gene and Additional Detections

A comparison of resistance genes detected using the FA and the corresponding AST results is presented in Supplementary Table S1. Among the 15 *S. aureus* cases detected only using FA and not culture, the *mecA/C* and *MREJ* genes were detected in 9 cases. Of the 9 *K. pneumoniae* cases detected only using FA, *CTX-M* was identified in 2 cases, and *KPC* was detected in 1 case. Additionally, *CTX-M* was detected by FA in 1 *K. pneumoniae* and 1 *E. coli* case, for which ESBL production was not identified by culture-based testing.

Ten bacterial species not included in the FA panel were detected via culture: *Achromobacter xylosoxidans* (*n* = 1), *Providencia stuartii* (*n* = 1), *Staphylococcus haemolyticus* (*n* = 1), and *Stenotrophomonas maltophilia* (*n* = 6). Viruses detected via FA included adenovirus (*n* = 2), coronavirus (*n* = 3), influenza A (*n* = 7), influenza B (*n* = 1), respiratory syncytial virus (*n* = 4), human rhinovirus/enterovirus (*n* = 5), and human metapneumovirus (*n* = 1). Among the 18 specimens in which viruses were detected, 10 also harbored bacterial pathogens. No atypical bacteria were detected.

## 4. Discussion

The diagnostic yield of multiplex PCR for bacterial detection using BW specimens has not been extensively studied. In this study, the FA performed on BW specimens demonstrated high PPA with conventional culture for bacterial identification, and the semi-quantitative results showed a moderate correlation. FA yielded a higher number of bacterial targets, with minimal false negatives compared to conventional culture. Moreover, FA demonstrated rapid and accurate detection of antimicrobial resistance genes.

BAL is a well-established method for identifying respiratory pathogens and minimizes contamination from upper airway flora [7,13]. However, BW offers a less invasive and more accessible alternative, allowing specimen collection with reduced patient burden. In contrast, BAL carries potential procedural risks and should be used judiciously [14]. In many clinical situations, the accessibility of BW may outweigh the risks associated with BAL, making BW a practical alternative [8]. BW has also been shown to be useful in other molecular applications, such as cytomegalovirus (CMV) viral load monitoring in hematologic patients [10], and viral detection by PCR using BW specimens has shown high concordance with BAL (>90% concordance) [9]. Additionally, studies have suggested that the diagnostic yield of BW for detecting nontuberculous mycobacteria is comparable to that of BAL [15].

Unlike BAL and endotracheal aspirates, BW specimens are typically reported semi-quantitatively because established CFU/mL thresholds are lacking, which complicates the interpretation of FA semi-quantitative results. This absence of a standardized cutoff may also contribute to the detection of colonizing rather than pathogenic organisms. Previous studies using FA with BAL specimens have shown that the correlation between FA and culture improves as bacterial load increases, while lower bacterial loads show weaker agreement [5]. In our study, the implementation of a standardized BW protocol resulted in a moderate correlation between FA and culture. Most culture-negative specimens aligned with low FA bin values, although some showed high FA loads. Culture “slight” and “moderate” categories generally corresponded to 10^4^–10^5^ copies/mL FA bins, indicating acceptable concordance in bacterial burden estimation between the two methods. These findings suggest that FA’s semi-quantitative reporting can be meaningfully interpreted when applied to BW specimens. In contrast, discordant results between FA bin values and culture growth may be attributable to antibiotic-suppressed bacterial viability, PCR detection of nonviable organisms, or intrinsic differences between nucleic acid quantity and viable colony-forming units. In addition, culture results are highly influenced by the microbial matrix of the specimen and are more dependent on the host’s immune status. Culture-based testing is also influenced by laboratory-specific practices and interpretation by technologists [16]. Therefore, this was a single-center study, and given the absence of a universally standardized protocol for BW collection, the observed correlations should be interpreted with caution.

Currently, data regarding molecular diagnostic testing using BAL and BW specimens for the detection of pneumonia pathogens remain limited. A direct comparison of the two specimen types could help elucidate the impact of oropharyngeal contamination on false-positive results in molecular testing using BW specimens. Compared to BAL, BW specimens are generally considered to have lower diagnostic value in culture-based testing [17], primarily due to a higher risk of contamination with commensal flora [7,18]. This may be partly explained by findings showing that endotracheal aspirates, compared to BAL or mini-BAL, are three times more likely to yield ≥3 pathogens, raising concerns about the quantitative correlation of multiplex PCR with BW specimens [11]. In our study, many BW isolates were culture-negative but FA-positive. Most of these organisms were species commonly regarded as upper airway colonizers, and Table 2 shows that their FA bin values were generally low to moderate. A substantial proportion of these detections may reflect colonization rather than true infection, indicating that the high sensitivity of FA may also increase the detection of non-pathogenic or colonizing organisms in BW specimens. In addition, FA-only detections of pathogens such as *H. influenzae*, *S. aureus*, and *K. pneumoniae* may reflect prior antibiotic exposure, especially in elderly or COPD patients who frequently harbor these bacteria in the upper airway. In this context, among nine patients with COPD, FA detected pathogens in two cases despite negative culture results (*S. aureus*, *n* = 1; *S. pneumoniae*, *n* = 1). Nevertheless, culture-negative but FA high load (≥10^7^ copies/mL) may lead to clinical uncertainty. A retrospective review of medical records was conducted for the nine cases in which bacterial load was reported as ≥10^7^ copies/mL using FA despite negative culture results. These included *H. influenzae* (*n* = 3). *S. aureus* (*n* = 1), *S. agalactiae* (*n* = 2), and *S. pneumoniae* (*n* = 3). None of these organisms was identified in subsequent blood cultures or additional respiratory cultures, suggesting a lack of corroborating microbiological evidence of true infection. In a study evaluating FA, such common upper airway organisms may have uncertain clinical significance [19]. These findings suggest that upper airway colonizers may occasionally yield high FA loads without representing clinically meaningful lower respiratory tract infection. Because this study was retrospective and based on residual specimens, FA results were not reported to clinicians in real time. Therefore, the impact of FA results on antibiotic prescribing or clinical decision-making could not be evaluated. As the clinical implications of culture-negative but high FA loads have not been clearly established, BW-based FA results should be interpreted with caution, and treatment decisions should incorporate clinical context rather than molecular load alone.

Our laboratory does not routinely report normal oral flora, and if no clinically significant pathogens are isolated, the result is reported as “no pathogen”. Consequently, it may be difficult for clinicians to discern potential oropharyngeal contamination based solely on FA results. Although BAL specimens are less prone to contamination, they are not immune [20,21]. Because BW specimens are more susceptible to oropharyngeal contamination than BAL specimens, the growth of mixed commensal flora on culture plates can occasionally complicate plate interpretation and obscure the identification of clinically relevant pathogens. In addition, variations in culture media quality, specimen condition, and inherent methodological differences between culture-based and molecular assays may further contribute to discordant results between FA and culture. Therefore, further prospective studies are needed to evaluate the extent of upper airway contamination and to define appropriate FA semi-quantitative thresholds for BW compared with BAL.

FA demonstrated 100% PPA for eight targets; however, lower PPA values were observed for others, such as *E. cloacae* complex and *K. aerogenes*, suggesting potential limitations in sensitivity for certain organisms. Notably, *Enterobacter hormaechei*, which was identified by culture but not detected by FA, may be underreported due to a sequence variant affecting the primer, as acknowledged by the manufacturer [22]. Furthermore, the use of predefined detection thresholds of FA may lead to under-detection of pathogens with low bacterial burden near the limit of detection. Nevertheless, most targets demonstrated NPA values above 97%. The consistently high OPA suggests that FA may serve as a reliable alternative to conventional culture for pathogen identification in BW specimens, particularly in clinical settings where rapid results are essential. In addition, FA enhances diagnostic breadth by detecting atypical pneumonia pathogens and respiratory viruses. The use of multiplex panels has been shown to increase viral detection [19,23] and support antibiotic stewardship by enabling more targeted antimicrobial therapy [5]. The FA panel detected multiple respiratory viruses in this study. Viral detection co-occurred with bacterial pathogens in 10 of the 18 virus-positive specimens. Although BW specimens are less commonly used for viral infections, it is suggested that BW specimen-based FA may help identify alternative etiologies of pneumonia and reduce unnecessary antibiotic use. Although atypical bacteria were not detected in this study, identifying the causative agents of atypical pneumonia is inherently challenging. Therefore, the use of FA is a valuable option that may help prevent the delayed diagnosis of these pathogens. Nevertheless, FA is limited to its predefined target panel and may miss clinically important pathogens such as *S. maltophilia*; therefore, negative FA results should be interpreted with caution, and culture remains indispensable.

Moreover, FA typically provides results 1–2 days faster than conventional AST, offering value in regions with high antimicrobial resistance, where timely intervention is crucial [19]. In our study, a notable number of MRSA (methicillin-resistant *Staphylococcus aureus*) gene detections (*n* = 9) were observed in *S. aureus* cases that were negative by culture but detected only using FA. Among these cases, five patients did not undergo MRSA screening, while four were screened; of those, MRSA was not detected in two. Our laboratory utilizes chromogenic agar for MRSA screening. The sensitivity of chromogenic agar for MRSA is variable (70% to 95%) [24,25], whereas PCR-based screening offers a faster turnaround time and shorter isolation periods [26]. In our cohort of predominantly elderly and critically ill patients, the additional resistance information provided using FA may support antibiotic decision-making and infection control.

This study has several limitations. First, culture alone was used as the reference standard, which may introduce false-negative bias, particularly in the context of prior antibiotic exposure. Second, this was a single-center study that predominantly included elderly patients, which may limit the generalizability of the findings. Third, the absence of established quantitative thresholds for BW specimens complicates the interpretation of both culture and FA results, and antibiotic exposure was not systematically evaluated.

Our study uniquely compared FA and culture simultaneously using the same BW specimens without processing delay. Under identical preanalytical conditions, FA detected more pathogens than culture, with minimal false negatives. Rapid detection of pathogens and resistance genes by FA may enable earlier optimization of antimicrobial therapy, particularly when culture results are pending or negative. However, because BW specimens are more prone to detecting colonizing organisms, FA findings should be interpreted in conjunction with the clinical context. When BAL is not feasible, FA testing on BW specimens may represent a practical alternative, offering semi-quantitative data that can support clinical decision-making.

## Figures and Tables

**Table 1:** Cross-tabulation of the number of pathogens detected using conventional culture and FilmArray Pneumonia Panel.

Number of Conventional Culture Detected Pathogens	Number of FilmArray Pneumonia Panel Detected Pathogens							Total
	0	1	2	3	4	5	6	
0	90	18	3					111
1	3	42	14	1	3		1	64
2		1	5	2	1	1	1	11
3				1	1	2		4
Total	93	61	22	4	5	3	2	

**Table 2:** Semi-quantitative comparison of bacterial load between FilmArray Pneumonia Panel and conventional culture.

Culture	FilmArray Pneumonia Panel				
	Not Detected	10^4^	10^5^	10^6^	≥10^7^
Not Detected	91	36	14	13	9
		ABA (*n* = 2) ECL (*n* = 1) ECO (*n* = 4) HIN (*n* = 4) KAE (*n* = 1) KPN (*n* = 5) MCA (*n* = 1) PRO (*n* = 2) PAE (*n* = 3) SMA (*n* = 1) SAU (*n* = 7) SAG (*n* = 2) SPN (*n* = 3)	ABA (*n* = 1) ECL (*n* = 1) HIN (*n* = 1) KPN (*n* = 3) PAE (*n* = 2) SAU (*n* = 3) SAG (*n* = 3)	ABA (*n* = 1) HIN (*n* = 2) KPN (*n* = 1) PAE (*n* = 1) SAU (*n* = 4) SAG (*n* = 2) SPN (*n* = 2)	HIN (*n* = 3) SAU (*n* = 1) SAG (*n* = 2) SPN (*n* = 3)
Slight	4	13	13	8	2
	KPN (*n* = 2) PAE (*n* = 2) *E. hormaechei* (*n* = 1)	ABA (*n* = 1) KOX (*n* = 1) KPN (*n* = 9) SAU (*n* = 2)	ABA (*n* = 2) ECL (*n* = 1) ECO (*n* = 3) KAE (*n* = 1) KOX (*n* = 1) KPN (*n* = 1) PAE (*n* = 1) SAU (*n* = 3)	ABA (*n* = 1) ECO (*n* = 1) PAE (*n* = 2) SMA (*n* = 1) SAU (*n* = 3)	ABA (*n* = 1) PAE (*n* = 1)
Moderate	1	9	9	10	8
	KAE (*n* = 1)	ECL (*n* = 2) ECO (*n* = 1) KAE (*n* = 1) KPN (*n* = 2 PRO (*n* = 1) PAE (*n* = 1) SAU (*n* = 1)	ABA (*n* = 1) ECO (*n* = 1) KPN (*n* = 1) PAE (*n* = 2) SAU (*n* = 4)	ABA (*n* = 2) KAE (*n* = 1) KPN (*n* = 1) PAE (*n* = 2) SMA (*n* = 1) SAU (*n* = 3)	ABA (*n* = 1) KPN (*n* = 1) PAE (*n* = 2) SAU (*n* = 3) SPN (*n* = 1)
Heavy		1	4	4	12
		KPN (*n* = 1)	ECO (*n* = 1) KAE (*n* = 1) KPN (*n* = 1) SAU (*n* = 1)	ABA (*n* = 2) ECO (*n* = 1) KPN (*n* = 1)	ABA (*n* = 5) ECO (*n* = 1) HIN (*n* = 1) KPN (*n* = 1) PAE (*n* = 3) SAU (*n* = 1)

Abbreviations: ABA, *A. baumannii*; ECL, *E. cloacae complex*; ECO, *E. coli*; HIN, *H. influenzae*; KAE, *K. aerogenes*; KOX, *K. oxytoca*; KPN, *K. pneumoniae*; MCA, *M. catarrhalis*; PAE, *P. aeruginosa*; SMA, *S. marcescens*; SAU, *S. aureus*; SAG, *S. agalactiae*; SPN, *S. pneumoniae*.

**Table 3:** Diagnostic performance of the FilmArray Pneumonia Panel compared to conventional culture for each bacterial target.

Pathogens	Culture +		Culture −		PPA (95% CI)	NPA (95% CI)	OPA (95% CI)
	FA +	FA −	FA +	FA0020 −			
*A. baumannii*	15	0	4	171	100 (79.6–100)	97.7 (94.3–99.1)	97.9 (94.7–99.2)
*E. cloacae complex*	3	1	2	184	75.0 (30.1–95.4)	98.9 (96.2–99.7)	98.4 (95.5–99.5)
*E. coli*	9	0	4	177	100 (70.1–100)	97.8 (94.5–99.2)	97.9 (94.7–99.2)
*H. influenzae*	1	0	10	179	100 (20.7–100)	94.7 (90.5–97.1)	94.7 (90.6–97.1)
*K. aerogenes*	4	1	1	184	80.0 (37.6–96.4)	99.5 (97.0–99.9)	99.0 (96.2–99.7)
*K. oxytoca*	2	0	0	188	100 (34.2–100)	100 (98.0–100)	100 (98.0–100)
*K. pneumoniae*	19	2	9	160	90.5 (71.1–97.4)	94.7 (90.2–97.2)	94.2 (89.9–96.7)
*M. catarrhalis*	0	0	1	189	NA	99.5 (97.1–99.9)	99.5 (97.1–99.9)
*Proteus* spp.	1	0	2	187	100 (20.7–100)	98.9 (96.2–99.7)	99.0 (96.2–99.7)
*P. aeruginosa*	14	2	6	168	87.5 (64.0–96.5)	96.6 (92.7–98.4)	95.8 (91.9–97.9)
*S. marcescens*	2	0	1	187	100 (34.2–100)	99.5 (97.1–99.9)	99.5 (97.1–99.9)
*S. aureus*	21	0	15	154	100 (84.5–100)	91.1 (85.9–94.6)	92.1 (87.4–95.2)
*S. agalactiae*	0	0	9	181	NA	95.3 (91.2–97.5)	95.3 (91.2–97.5)
*S. pneumoniae*	1	0	8	181	100 (20.7–100)	95.8 (91.9–97.8)	95.8 (91.9–97.9)
*S. pyogenes*	0	0	0	190	NA	100 (98.0–100)	100 (98.0–100)

Abbreviations: +, positive; −, negative; FA, FilmArray Pneumonia Panel; PPA, positive percent agreement; NPA, negative percent agreement; OPA, overall percent agreement; CI, confidence interval; NA, not available.

## Data Availability

The data presented in this study are available upon request from the corresponding author.

## Supplementary Materials

The following supporting information can be downloaded at: https://www.mdpi.com/article/10.3390/diagnostics16010091/s1, Table S1: A comparison of resistance genes detected by the FA and the corresponding AST results.
